# 222 nm ultraviolet radiation C causes more severe damage to guard cells and epidermal cells of *Arabidopsis* plants than does 254 nm ultraviolet radiation

**DOI:** 10.1007/s43630-021-00123-w

**Published:** 2021-11-03

**Authors:** Momo Otake, Kaoru Okamoto Yoshiyama, Hiroko Yamaguchi, Jun Hidema

**Affiliations:** 1 Department of Molecular and Chemical Life Sciences, Graduate School of Life Sciences, Tohoku University, Sendai, 980-8577 Japan; 2 Division for the Establishment of Frontier Sciences of the Organization for Advanced Studies, Tohoku University, Sendai, 980-8577 Japan

## Abstract

Lamps that emit 222 nm short-wavelength ultraviolet (UV) radiation can be safely used for sterilization without harming human health. However, there are few studies on the effects of 222 nm UVC (222-UVC) radiation exposure on plants compared with the effects of germicidal lamps emitting primarily 254 nm UVC (254-UVC) radiation. We investigated the growth inhibition and cell damage caused by 222-UVC exposure to *Arabidopsis* plants, especially mitochondrial dynamics, which is an index of damage caused by UVB radiation. Growth inhibition resulted from 254-UVC or 222-UVC exposure depending on the dose of UVC radiation. However, with respect to the phenotype of 222-UVC-irradiated plants, the leaves curled under 1 kJ m^−2^ and were markedly bleached under 10 kJ m^−2^ compared with those of plants irradiated with 254-UVC. The cellular state, especially the mitochondrial dynamics, of epidermal and mesophyll cells of *Arabidopsis* leaves exposed to 254-UVC or 222-UVC radiation was investigated using *Arabidopsis* plants expressing mitochondrial matrix-targeted yellow fluorescent protein (MT-YFP) under the control of Pro35S to visualize the mitochondria. 222-UVC (1 or 5 kJ m^−2^) severely damaged the guard cells within the epidermis, and YFP signals and chloroplast autofluorescence in guard cells within the epidermis exposed to 222-UVC (1 or 5 kJ m^−2^) were not detected compared with those in cells exposed to 254-UVC radiation. In addition, 222-UVC irradiation led to mitochondrial fragmentation in mesophyll cells, similar to the effects of 254-UVC exposure. These results suggest that 222-UVC severely damages guard cells and epidermal cells and that such damage might have resulted in growth inhibition.

## Introduction

Germicidal lamps that emit primarily 254 nm ultraviolet (UV) C have been utilized for sterilization and virus inactivation, because this wavelength is effective for killing bacteria. However, it is well known to be harmful to the skin and eyes, causing erythema and keratitis, respectively [1–3]. In recent years, 207 nm krypton–bromine and 222 nm krypton–chlorine excimer lamps have attracted attention for their ability to emit radiation that effectively inactivates a wide range of microorganisms, including bacteria, bacterial spores, and viruses, and that does not endanger human health [4–6]. The main factor by which 254 nm UVC (254-UVC) endangers human health is the formation of pyrimidine dimers, such as cyclobutane pyrimidine dimers (CPDs) or pyrimidine–pyrimidone (6–4) photoproducts (6–4 PPs) [7–9]. However, UVC radiation with shorter wavelengths does not penetrate deep into epidermal cells: for example, 222 nm UVC radiation (222-UVC) does not reach the nuclei of murine epidermal cells (≥ 10 μm) but does reach bacterial nuclei (≤ 1 μm) [10, 11]. Recently, many reports have experimentally shown that shorter wavelength UVC lamps (those that emit 207 or 222 nm UVC) can be safely used for sterilization without harming human health [12–14].

The leaf structure of plants varies widely among plant species, but epidermal cells are 0.1–1 μm thick. In addition, in some plant species, guard cells, which are important for gas exchange, are also present among the epidermal cells of the leaves. Therefore, short-wavelength UVC light may cause severe damage to leaf epidermal cells. However, little is known about the action spectrum of plant damage and responses at shorter wavelength UVC-irradiated region, or the effect of irradiation of 222-UVC on the growth of or epidermal cell damage in plants. To understand the effects of high doses of 222-UVC irradiation on plants, we investigated the growth inhibition and cell damage caused by 222-UVC exposure. We previously demonstrated that a high dose of UVB radiation disrupts mitochondrial function and causes mitochondrial fragmentation in the mesophyll cells of *Arabidopsis* leaves, and that the accumulation of mitochondria damaged by UVB radiation in the cells contributes to UVB-induced growth inhibition [15]. In this study, to understand the effects of 222-UVC irradiation on *Arabidopsis* plant, 222-UVC-induced growth inhibition and the mitochondrial dynamics in the epidermis and mesophyll cells of *Arabidopsis* were investigated, compared with that of 254-UVC-irradiated *Arabidopsis*.

## Materials and methods

### Light sources

The UVC radiation used in this study was provided by a germicidal lamp (254-UVC) (Toshiba GL20; Toshiba Electric, Ltd., Tokyo, Japan) at a distance of 20 cm. The far-UVC source used in this study was a 15 W 222 nm KrCl excimer lamp (222-UVC) made by ORC Manufacturing Co., Ltd. (Tokyo, Japan), and 222-UVC was provided at a distance of 12.5 cm. The UVC irradiations were performed in an open dark space set at 23 °C. The spectra and energy fluence rates were measured with a spectroradiometer (USR-45DA; Ushio, Inc., Tokyo, Japan).

### UV survival assays for *Escherichia coli* and P1 phages

For *E. coli* UV survival assays, saturated cultures were used. Wild-type *E. coli* (AB1157) was grown overnight at 37 °C (~ 17–18 h) until saturation [10^9^ colony-forming units (CFU) mL^−1^] was reached. The cultures were subsequently diluted in Luria–Bertani (LB) broth, and 100 µL of different dilutions was plated on LB agar plates. These plates were then exposed to UV irradiation (254 nm or 222 nm) for different time periods. The 254-UVC and 222-UVC radiation source provided irradiation at rates of 7.1 and 1.7 J m^−2^ s^−1^, respectively. The samples were exposed to 254-UVC for 7 s (50 J m^−2^), 14 s (100 J m^−2^), and 21 s (150 J m^−2^), and exposed to 222-UVC for 29 s (50 J m^−2^), 59 s (100 J m^−2^), and 88 s (150 J m^−2^). After UV irradiation, the plates were covered with foil for shielding from light and placed in a 37 °C incubator. Unexposed samples were used as control plates. The number of CFU per milliliter was recorded the next day.

For P1 phage UV survival assays, plaque-forming activity was evaluated. Host bacteria (MG1655) were grown in LB media at 37 °C to an OD_600_ of 1.0. Suspensions of P1 phage [8 × 10^8^ plaque-forming units (PFU) mL^−1^] in Tris buffer in open Petri dishes were placed on an oscillating platform and stirred gently during irradiation. Samples were withdrawn and assayed for plaque formation. Diluted P1 phages were added to a culture of bacteria [multiplicity of infection (MOI) = 0.1], allowed to absorb for 20 min at 37 °C, and then plated. After irradiation, the samples were plated in soft agar and then incubated at 37 °C for 1 day. The number of PFU per milliliter was subsequently recorded. Each experiment was repeated three times, and the data are presented as the means ± SEs.

### Plant materials and growth conditions

A transgenic *Arabidopsis* line in the Columbia background harboring mitochondrial matrix-targeted yellow fluorescent protein (MT-YFP) driven by Pro35S was used [15]. The transgenic *Arabidopsis* line MT-YFP-WT was grown vertically on ½-strength Murashige and Skoog (MS) medium agar plates in chambers at 23 °C under a 16 h light/8 h dark photoperiod provided by white fluorescent lamps (140 μmol m^−2^ s^−1^). Seven-day-old seedlings were used for all experiments.

### UV treatment and measurement of plant growth

Seven-day-old seedlings were irradiated with a 0.15–10 kJ m^−2^ dose of 254-UVC or 222-UVC. Plants were exposed to the 254-UVC for 1 min and 3 s (500 J m^−2^), 2 min and 5 s (1000 J m^−2^), 5 min and 14 s (2500 J m^−2^), 10 min and 27 s (5000 J m^−2^), 15 min and 41 s (7500 J m^−2^), 20 min and 55 s (10,000 J m^−2^), and exposed to the 222-UVC for 5 min and 2 s (500 J m^−2^), 10 min and 4 s (1000 J m^−2^), 25 min and 10 s (2500 J m^−2^), 50 min and 19 s (5000 J m^−2^), 1 h, 15 min and 29 s (7500 J m^−2^), 1 h, 40 min and 39 s (10,000 J m^−2^). After UVC irradiation, the plants were returned to the growth chamber. The aboveground parts of the plants were cut off, and their fresh weights (FWs) were measured individually. At least 15 plants from three biological replications were analyzed.

### Laser‐scanning confocal microscopy (LSCM) imaging

LSCM imaging was performed using a C-Apochromat LD63 × water-immersion objective (numerical aperture = 1.15; LSM800, Carl Zeiss, Oberkochen, Germany). Fluorescence images of YFP (excitation at 488 nm; emission at 500–550 nm) and chlorophyll autofluorescence (excitation at 640 nm; emission at 650–700 nm) were subsequently obtained [15].

### Measurement of mitochondrial number and volume

Transgenic *Arabidopsis* lines harboring MT-YFP were irradiated with 254- or 222-UVC. Twenty-four hours after UVC exposure, the plants were observed via LSCM. Fixed image areas (212 × 212 × 40 μm each) were monitored by changing the focus during LSCM. The Z-stack images from LSCM were converted to three-dimensional pictures, and the number and volume of mitochondria were measured using Imaris microscopy analysis software (Bitplane, Zurich, Switzerland) [15]. To determine the number and volume of mitochondria, three plants at each timepoint were analyzed [*n* = 3 (≥ 5 cells)].

## Results and discussion

**Fig. 1:**
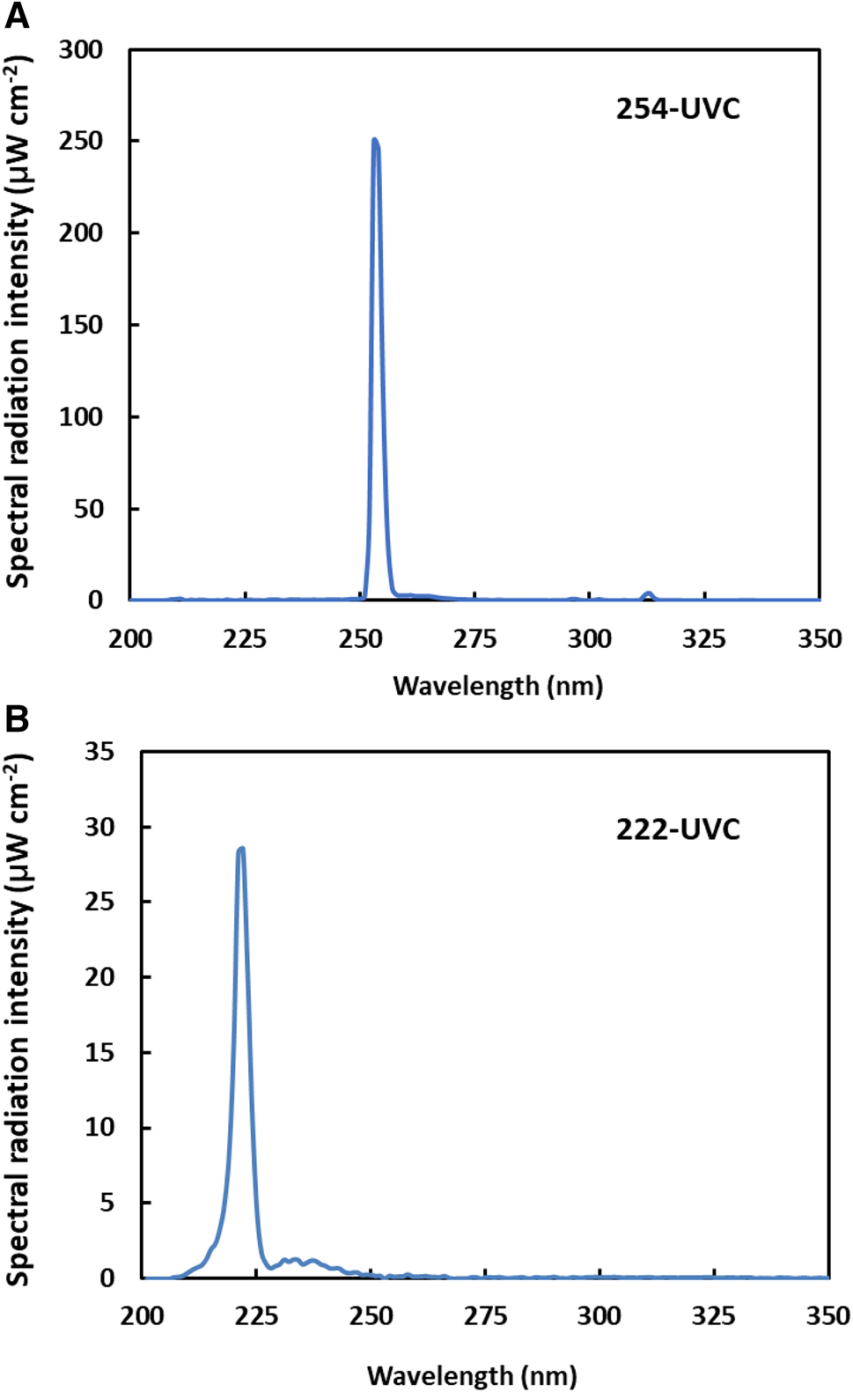
Spectra of the light sources used in this study. **A** Spectra of a germicidal lamp (254-UVC) (Toshiba GL20; Toshiba Electric, Ltd.) at a distance of 20 cm. **B** Spectra of a 15 W 222 nm KrCl excimer lamp (222-UVC) made by ORC Manufacturing Co., Ltd. (Tokyo, Japan), at a distance of 12.5 cm. The 254-UVC and 222-UVC radiation sources emitted radiation at rates of 7.1 and 1.7 J m^−2^ s^−1^, respectively

The spectra of the 254-UVC and 222-UVC light sources used in this study are shown in Fig. 1. The 254-UVC light emits a dominant line spectrum from 250 to 270 nm, with a peak at 254 nm, and two small line spectra at 313 and 366 nm (Fig. 1A). The 222-UVC light source emitted a dominant line spectrum from 200 to 230 nm, with a peak at 222 nm, and a continuous spectrum from 230 to 265 nm, with peaks at 238 and 258 nm (Fig. 1B). The integrating intensity of the 235 to 265 nm UV is 9.0% of the integrating intensity of the 235–265 nm UV. Several studies investigating the effects of 222-UVC light on biological organisms have been performed in which light emitted from an excimer lamp source with 235–280 nm light was removed by the use of filters that restricted the emissions to 200–230 nm UV [14]. In this study, the light emitted from the excimer lamp was directly used without using a filter.

**Fig. 2:**
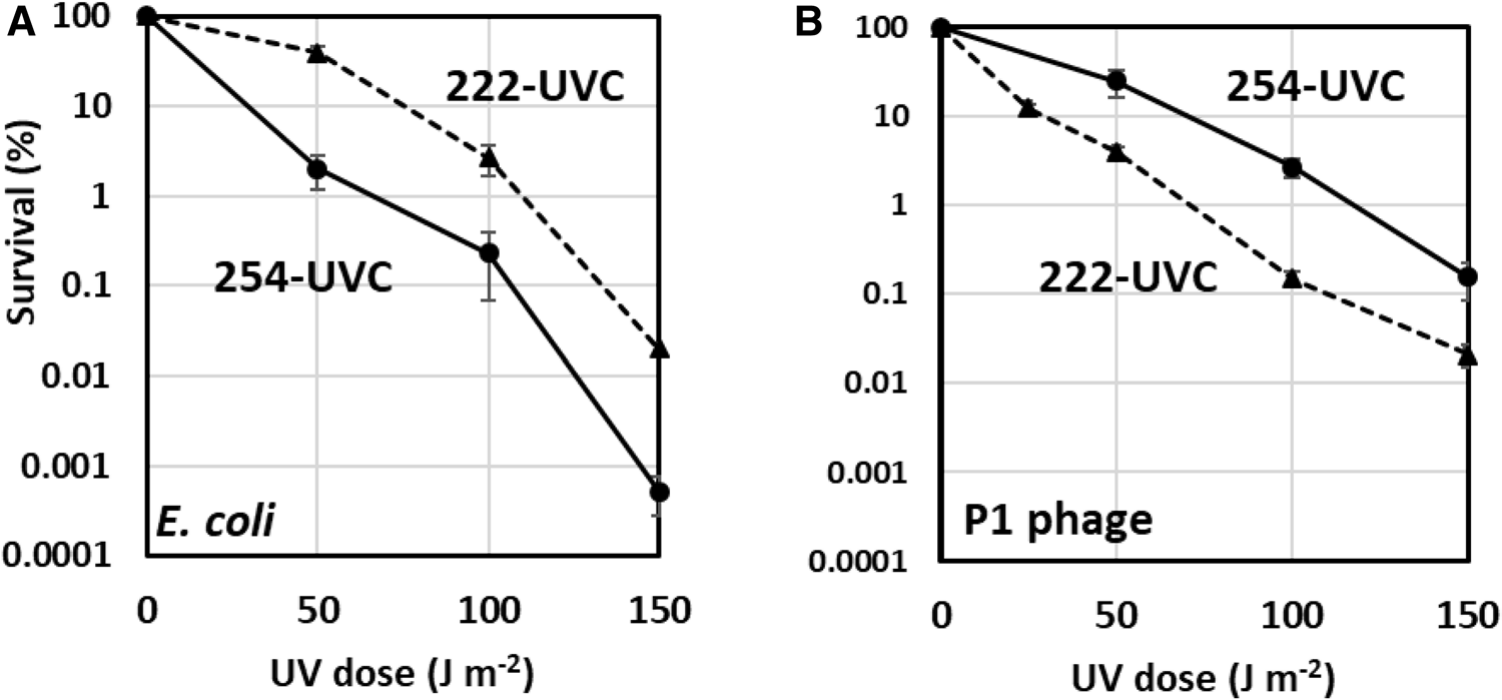
Survival curves of *E. coli* and P1 phages in response to UVC irradiation. *E. coli* wild-type strain AB1157 (**A**) and P1 phages (**B**) were irradiated with different doses of 254 nm (●) and 222 nm (▲) UVC. The values are the means of at least three experiments. The error bars indicate the SEs of three biological replicates

First, to confirm the characteristics of the effects of the 222-UVC radiation used in this study on biological organisms, the effects on the viability of *E. coli* and P1 phages, which have been widely reported thus far [16–19], were investigated. *E. coli* and P1 phages were subjected to a different dose (0–150 J m^−2^) of 254-UVC or 222-UVC radiation, and the survival rate of *E. coli* (Fig. 2A) or P1 phages (Fig. 2B) in response to each UVC radiation dose was measured. *E. coli* and P1 phage survival decreased with increasing dose of 254-UVC or 222-UVC. However, the susceptibility of *E. coli* and P1 phages differed in response to 254-UVC and 222-UVC; *E. coli* was more sensitive to 254-UVC than to 222-UVC, whereas P1 phages were more sensitive to 222-UVC than to 254-UVC. The 99% lethal dose (LD99) for *E. coli* disinfection by 254-UVC and 222-UVC was 65 and 110 J m^−2^, respectively. In contrast, the LD99 for P1 phage disinfection by 254-UVC and 222-UVC was 117 and 70 J m^−2^, respectively. When comparing susceptibility to 254-UVC and susceptibility to 222-UVC, it has been reported that *E. coli* was more susceptible to 254-UVC than to 222-UVC [16]. In contrast, MS2 bacteriophages were more sensitive to 222-UVC than to 254-UVC and were inactivated in response to a lower UV dose of 222-UVC; to reach 3log_10_ inactivation of MS2 bacteriophages, a more than twofold dose of 254-UVC (45 J m^−2^) compared with 222-UVC (20 J m^−2^) was needed [17]. Therefore, it was confirmed that the effects of the UVC light sources used in this study on the survival and inactivation of *E. coli* and phages were in accordance with the results reported thus far.

**Fig. 3:**
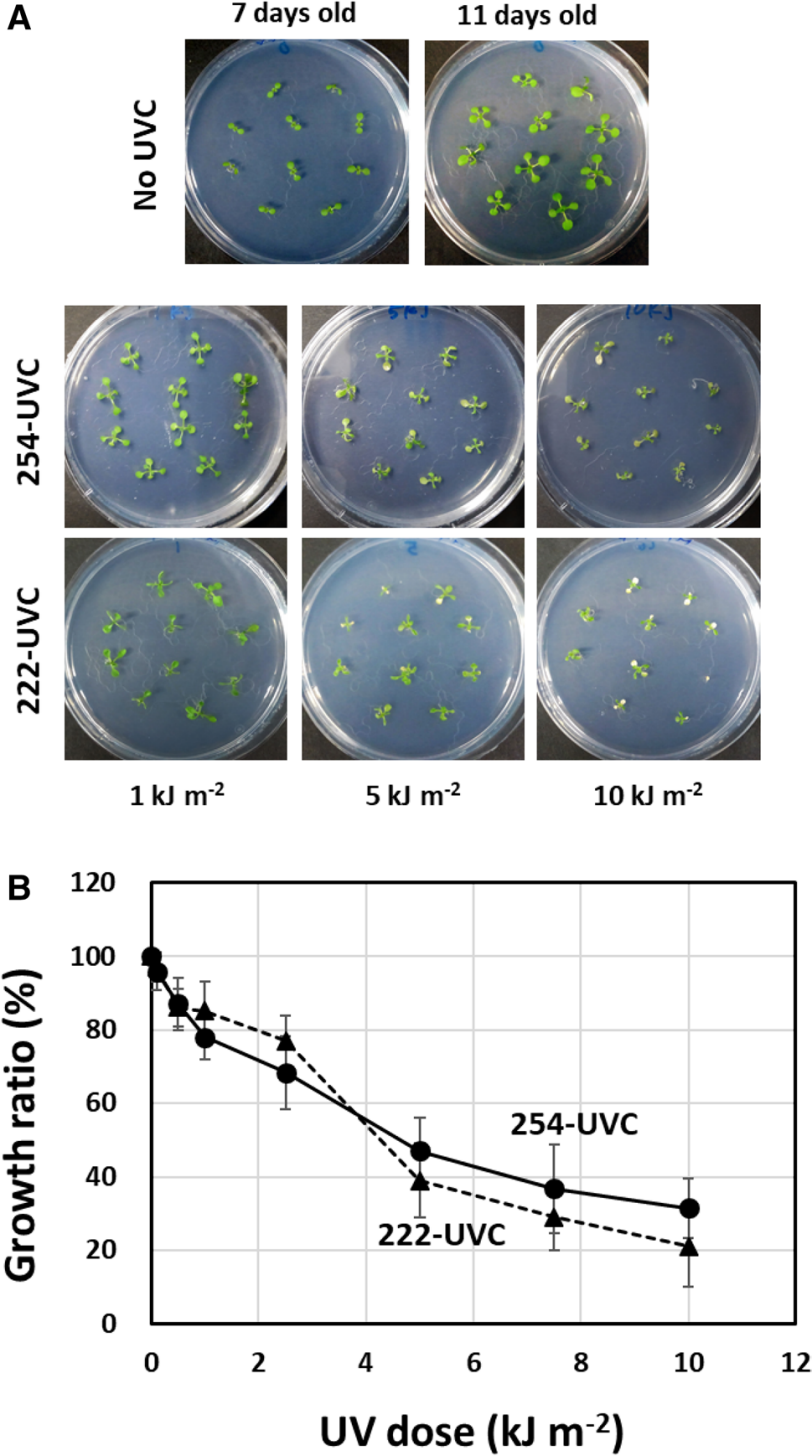
Effects of 254-UVC radiation or 222-UVC radiation on the growth of *Arabidopsis* plants. **A** Images of 11-day-old *Arabidopsis* plants exposed to UVC radiation. Seven-day-old seedlings were exposed to different doses of 254-UVC (●) or 222-UVC (▲) (0.1–10 kJ m^−2^), after which the plants were returned to the growth chamber. **B** The growth ratio, which is the ratio of irradiated aboveground FW to unirradiated aboveground FW, in response to 254-UVC or 222-UVC exposure

Next, using these UVC light sources, we analyzed the effects of 222-UVC on *Arabidopsis* plants and compared them with those of 254-UVC. Seven-day-old seedlings were exposed to different doses of 254-UVC or 222-UVC (0.1–10 kJ m^−2^), after which the plants were returned to the growth chamber. The growth damage phenotypes (shoot FW) were examined 4 days later (Fig. 3). The growth ratio, which was calculated as the ratio of irradiated aboveground FW to nonirradiated aboveground FW, in response to 254-UVC or 222-UVC exposure decreased depending on the dose of UVC radiation. There was no significant difference in the growth ratio depending on the UVC dose between 254-UVC and 222-UVC. However, with respect to the phenotype of 222-UVC-irradiated plants, the leaves curled under 1 kJ m^−2^ and were markedly bleached under 10 kJ m^−2^ compared with those of plants irradiated with 254-UVC. These results indicate that the effects of damage to plants might be different between 254-UVC and 222-UVC exposure, although there was no significant difference in the growth ratio curves for 254-UVC and 222-UVC.

**Fig. 4:**
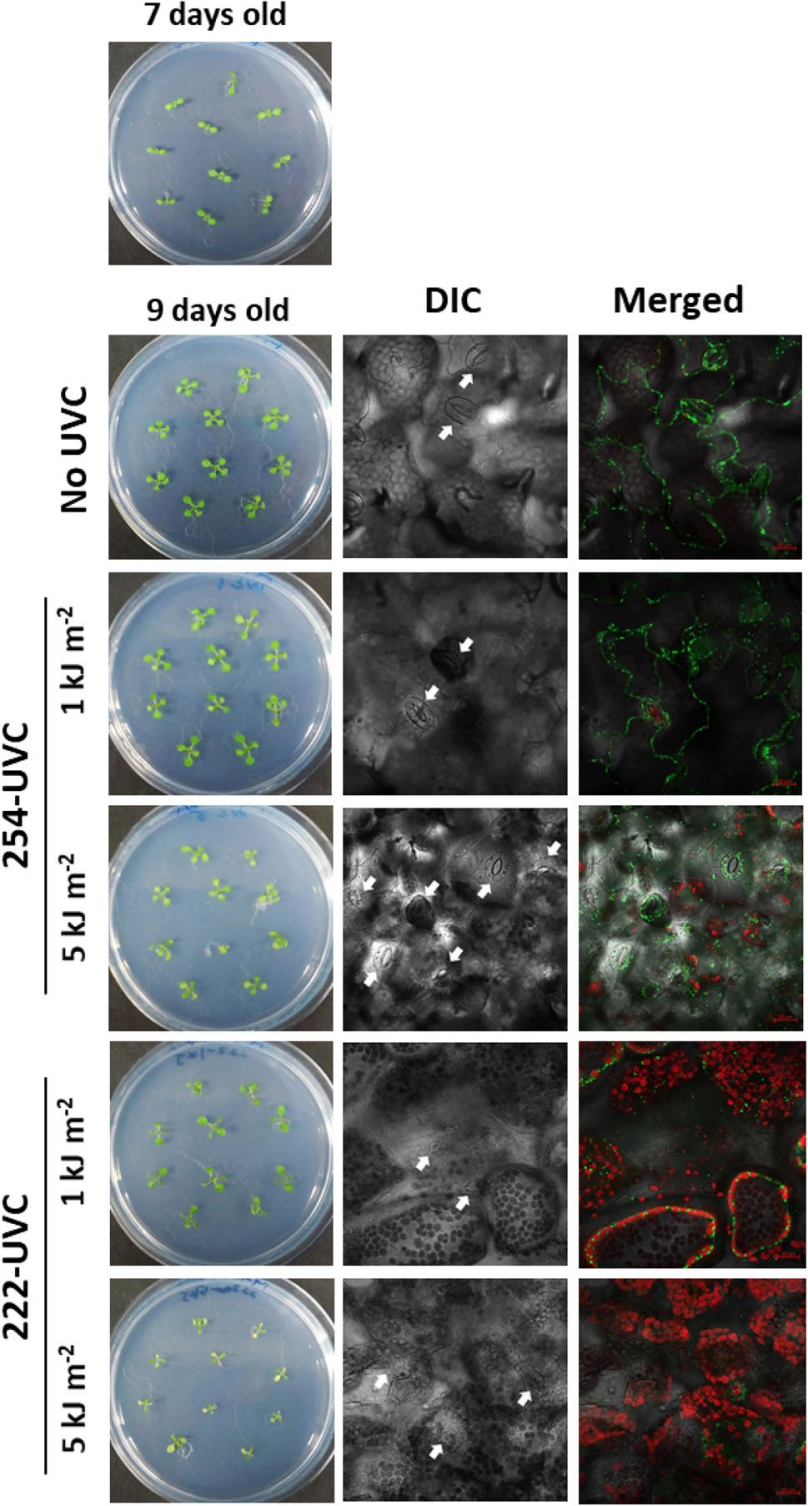
*Arabidopsis* plants and DIC and fluorescence images of the leaf epidermis observed via LSCM 24 h after 1 or 5 kJ m^−2^ 254-UVC or 222-UVC radiation. Scale bars, 20 μm. The white arrowheads indicate guard cells. Green or red signals indicate mitochondria or chloroplast, respectively

The effects of 254-UVC or 222-UVC irradiation were observed 4 days after irradiation, but the phenotypic effects of such damage were already visible 2 days after irradiation: 2 days after UVC irradiation, the plants exposed to 222-UVC were clearly more severely impaired than were those exposed to 254-UVC. These results indicate that 222-UVC induced more severe damage to cells at an early stage immediately after UVC irradiation. We previously demonstrated that a high dose of UVB radiation led to inactivation/fragmentation of mitochondria or damaged chloroplasts, which were removed by UVB-activated mitophagy [15] or chlorophagy [20], respectively. In addition, we showed that autophagy-deficient *Arabidopsis thaliana* mutants exhibit a UVB-sensitive phenotype similar to that of CPD-specific photolyase (PHR1)-deficient mutants [15, 20]. In addition to UVB-induced CPDs, UVB-induced damage to organelles, mitochondria, and chloroplasts is one of the principal causes of UVB-induced growth inhibition. Therefore, to investigate what kind of damage occurred in the cells after each UVC irradiation dose, the state inside the cells and the mitochondrial and chloroplast dynamics 24 h after each UVC irradiation dose were evaluated. In this experiment, *Arabidopsis* plants expressing MT-YFP under the control of Pro35S were used to visualize the mitochondria [15]. Figure 4 shows differential interference contrast (DIC) and fluorescence images of the leaf epidermis via LSCM at 24 h after each UVC irradiation dose. Guard cells are clearly observed in the epidermis of non-UV-irradiated leaves and leaves irradiated with 1 or 5 kJ m^−2^ 254-UVC. YFP signals and chloroplast autofluorescence were also detected in the guard cells of non-UV-irradiated leaves and leaves irradiation with 1 or 5 kJ m^−2^ 254-UVC. In contrast, in epidermal cells irradiated with 1 or 5 kJ m^−2^ 222-UVC, most guard cells were deformed, and YFP signals and chloroplast autofluorescence were not detected in these guard cells. In addition, although mesophyll cells cannot normally be observed when epidermal cells are observed, YFP signals and chloroplast autofluorescence of mesophyll cells were detected in the leaves irradiated with 1 or 5 kJ m^−2^ 222-UVC. Taken together, these results indicate that 222-UVC severely damages epidermal cells, including guard cells, and disrupts their function.

**Fig. 5:**
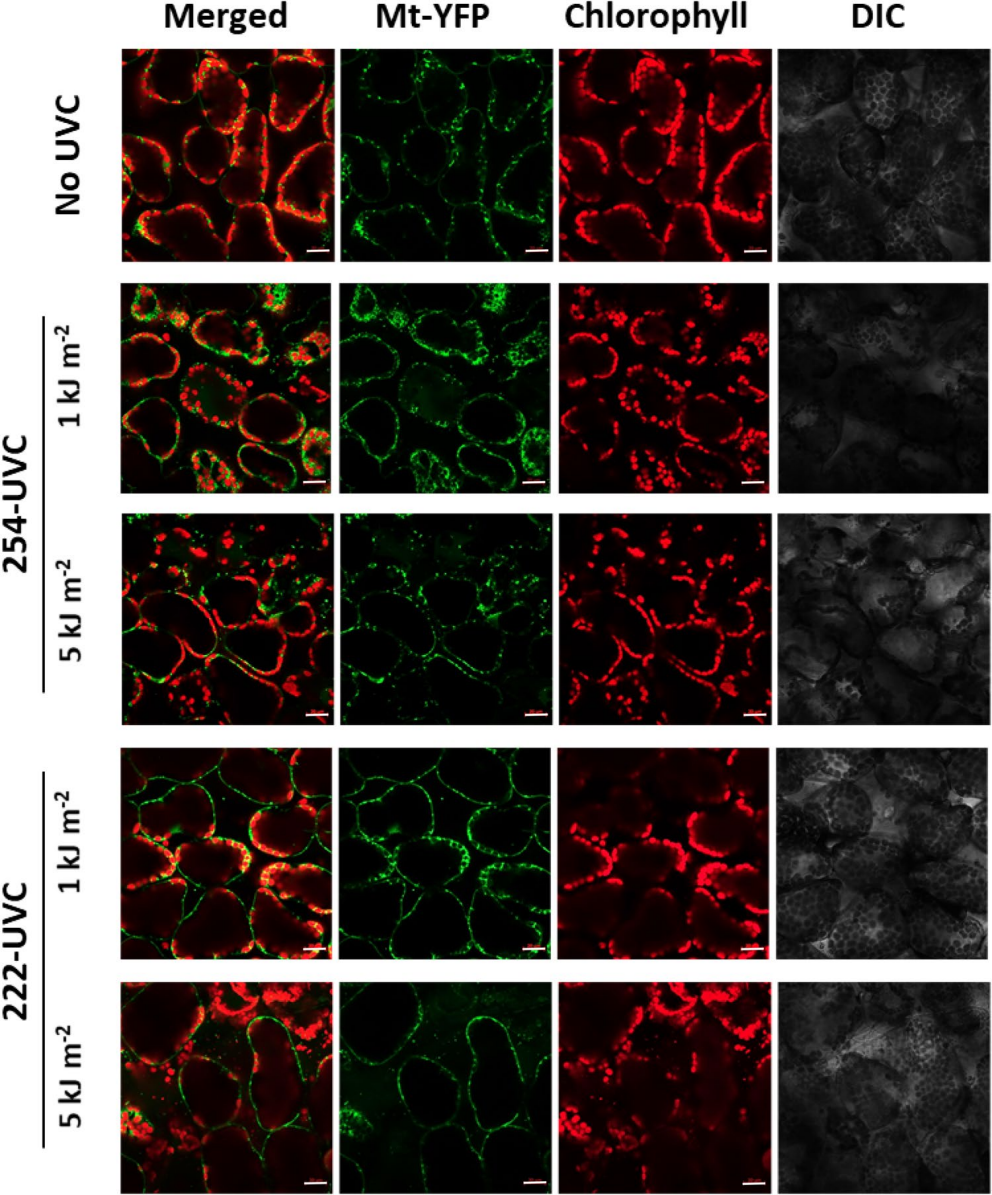
LSCM images of leaf mesophyll cells in MT‐YFP-expressing *Arabidopsis* seedlings (Mt-WT) that were not irradiated or exposed to 254-UVC or 222-UVC (1 and 5 kJ m^−2^). Images were taken 24 h after each UVC treatment. Green, mitochondria; red, chlorophyll. Scale bars, 20 μm

**Fig. 6:**
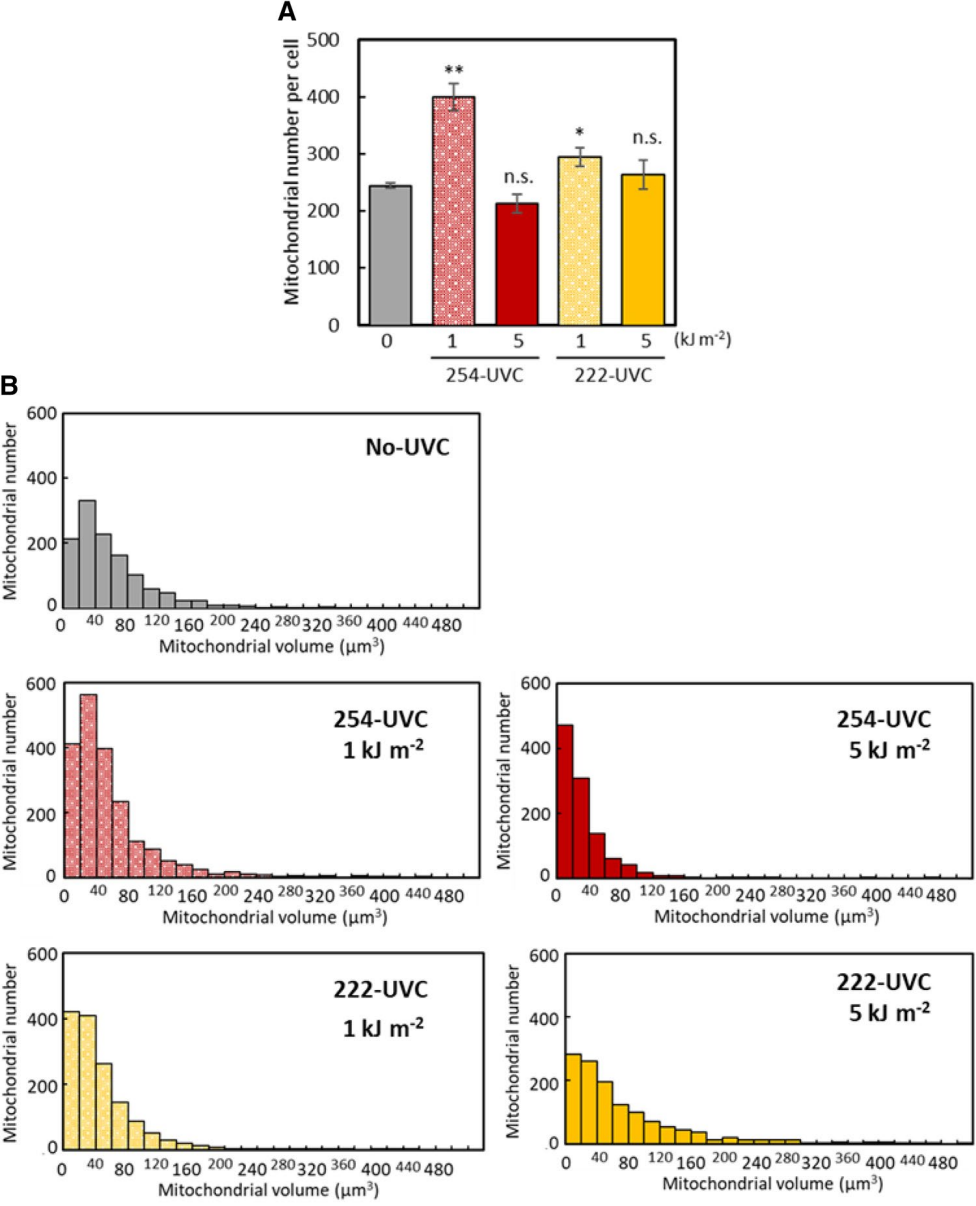
Changes in the number and volume of mitochondria in *Arabidopsis* exposed to 254-UVC or 222-UVC (1 and 5 kJ m^−2^). **A** The number of mitochondria (MT-YFP dots) per cell was counted with Imaris microscopy analysis software (Imaris software). Images from three different biological replicates were used for calculations. The data represent the means ± SEs (*n* ≥ 5 cells). The asterisks denote significant differences based on two-way ANOVA (n.s.: not significant, *p < 0.05, **p < 0.01). **B** Volume of each mitochondrion as estimated by three-dimensional conversion of MT-YFP dots by Imaris software

Next, the state of the mesophyll cells in the leaves irradiated with 254-UVC or 222-UVC was observed. We previously demonstrated that a high dose of UVB radiation can damage mitochondria and cause mitochondrial fragmentation in the mesophyll cells of *Arabidopsis* leaves and that these damaged dysfunctional mitochondria are removed by autophagy within 24 h after UVB irradiation [15]. Therefore, we investigated the effects of mitochondrial dynamics on the mesophyll cells of leaves irradiated with 254-UVC or 222-UVC. Figure 5 shows fluorescence images via LSCM of the intermediate layer of leaf mesophyll cells observed 24 h after each UVC irradiation. Within the mesophyll cells of the nonirradiated leaves, chloroplast autofluorescence was detected inside the cell wall, and YFP signals were uniformly detected at the edge of chloroplasts. Comparing the cells of the leaves irradiated with 1 kJ m^−2^ 254 UVC or 222-UVC with the cells of nonirradiated leaves, we found that the number of chloroplasts residing against the edge of the cell wall is decreased. In addition, YFP signals were often detected at a location away from the chloroplasts in the cells. Fixed image areas were monitored by changing the focus during LSCM. The Z-stack images from LSCM were converted to three-dimensional images, after which the number and volume of YFP fluorescent dots per cell (hereafter, “number of mitochondria”) were detected via Imaris microscopy analysis software. Figure 6 shows the number and volume of mitochondria in the cells exposed to 254- or 222-UVC radiation. The number of mitochondria in the cells significantly increased at 24 h after 1 kJ m^−2^ 254-UVC or 222-UVC exposure compared with no UVC irradiation exposure (Fig. 6A); furthermore, the number of small mitochondria (40 μm^−3^ or less in volume) is significantly increased at 24 h after 1 kJ m^−2^ 254-UVC or 222-UVC exposure. These results strongly indicate that the shorter wavelength UVC, like UVB exposure, can damaged mitochondria and caused mitochondrial fragmentation. On the other hand, in cells irradiated with 5 kJ m^−2^ 254 UVC, the placement or number of chloroplasts or mitochondria in cells was not uniform between cells (Fig. 5), some cells with decreased amounts of YFP signals and chloroplast autofluorescence were observed, and the cellular chloroplast or mitochondrial placement and numbers in cells irradiated with 5 kJ m^−2^ 222-UVC were uneven compared with those of observed cells irradiated with 5 kJ m^−2^ 254 UVC (Fig. 5). Comparing the number and volume of mitochondria in the cells irradiated with 5 kJ m^−2^ 254-UVC with those in the cells of nonirradiated leaves, we found that the number of small mitochondria (volume of 40 μm^−3^ or less) in cells irradiated with 5 kJ m^−2^ 254 UVC was higher than that in nonirradiated cells, while there was no significant difference in the number of mitochondria in cells (Fig. 6B). On the other hand, irradiation with 5 kJ m^−2^ 222-UVC appeared to severely damage mesophyll cells (Fig. 6B). Although there was no difference in the number of mitochondria in the irradiated cells compared to the nonirradiated cells, the number of small mitochondria (< 40 μm^−3^ in volume) tended to increase. We previously demonstrated that UVB treatment (1.5 W m^−2^ for 1 h) increased the number of mitochondria per cell while decreasing the volumes of individual mitochondria in the cells, meaning that UVB radiation led to the inactivation and fragmentation of mitochondria [16]. Therefore, these results indicated that 222-UVC irradiation led to the inactivation and fragmentation of mitochondria in mesophyll cells, similar to the effects of 254-UVC exposure.

## Conclusion

Today, attention is being paid to the use of 222 nm low-wavelength UV radiation (provided by 222 nm KrCl excimer lamps), which has low intracellular transmittance, as a technique for inactivating coronaviruses. A number of reports have investigated the effects of 222 nm lamp irradiation on *E. coli*, viruses, animals, cultured human cells, etc., and the number of reports is rapidly increasing. Since there are many reports experimentally, showing that a short-wavelength (222 nm) UVC lamp can be safely used for sterilization without harming human health, devices that use a 222-UVC lamp have been developed and are already in use. However, there are few studies on the effects of 222-UVC exposure on plants compared with the effects of 254-UVC exposure. Our results clearly show that irradiation of plants with 222-UVC or 254-UVC causes similar growth inhibition, although the mechanisms that cause growth inhibition are different. In particular, 222-UVC causes severe damage to guard cells and epidermal cells, and such damage might have resulted in growth inhibition.

In this study, we focused on the effect of shorter wavelength of 222-UVC irradiation on plant growth, especially 222-UVC-induced damage, negative responses. However, UV radiation, such as 254-UVC or UVB radiation, do not merely cause negative effects on living organisms, but induce various positive effects and responses, such as hormetic dose–response phenomenon characterized by a low-dose stimulation [21, 22]. For example, a low dose of UVC or UVB irradiation stimulates plant-defense responses or improves the quality of production in fruits [23–26]. Therefore, shorter wavelength of 222-UVC irradiation might cause not only negative responses but also a new positive and beneficial responses for living organisms. However, research on the effects of short-wavelength UVC radiation on living organisms, especially on plants, has just begun. To actually use devices that use a short-wavelength UVC lamp, more detailed analysis is required for various organisms, such as differences in response to irradiation intensity and duration of exposure.
